# Full characterization of superradiant pulses generated from a free-electron laser oscillator

**DOI:** 10.1038/s41598-023-33550-z

**Published:** 2023-04-18

**Authors:** Heishun Zen, Ryoichi Hajima, Hideaki Ohgaki

**Affiliations:** 1 Institute of Advanced Energy, Kyoto University, Gokasho Uji, Kyoto, 611-0011 Japan; 2 National Institutes for Quantum Science and Technology, Kizugawa, Kyoto, 619-0215 Japan

**Keywords:** Free-electron lasers, Ultrafast lasers

## Abstract

The detailed structure of superradiant pulses generated from a free-electron laser (FEL) oscillator was experimentally revealed for the first time. Owing to the phase retrieval with a combination of linear and nonlinear autocorrelation measurements, we successfully reconstructed the temporal waveform of an FEL pulse including its phase variation. The waveform clearly exhibits the features of a superradiant pulse, the main pulse followed by a train of sub-pulses with π-phase jumps, reflecting the physics of light-matter resonant interaction. From numerical simulations, the train of sub-pulses was found to originate from repeated formation and deformation of microbunches accompanied with a temporal slippage of the electrons and light field, a process quite different from coherent many-body Rabi oscillations observed in superradiance from atomic systems.

## Introduction

Superradiance (SR), originally predicted by Dicke in 1954^1^, is a quantum optical process in an N-body system coupled by a common electromagnetic field, in which the system exhibits accelerated radiative decay, generating a pulse of coherent light whose peak intensity is ∝ N^2^ and pulse duration is ∝ 1/N. Since Dicke’s first paper, many theoretical and experimental works have been conducted on SR in gases^2–8^ and solids^9–12^.

The first observation of SR was reported with optically pumped HF gas at a wavelength of 84 μm ^2^. In the experiment, ringing following the main pulse was observed in the SR pulse in addition to confirmation of the N^2^ dependence of the peak intensity. The ringing was later attributed to coherent Rabi oscillations similar to those predicted by Burnham and Chiao^13,14^; thus, the phenomenon is termed Burnham-Chiao ringing (BC ringing). BC ringing is a visible manifestation of the light-matter resonant interaction and is derived as a solution of the optical Bloch equations, which are the basis for the study of the light-matter resonant interaction to describe SR, photon echo, self-induced transparency, optical free induction decay, and more^15^. Coherent Rabi oscillations are also an important research subject for quantum information technology such as quantum processing in mesoscopic atomic ensembles^16^ and a spin-ensemble quantum memory^17^.

In the study of a free-electron laser (FEL), Bonifacio and Casagrande first predicted in 1985 that a single-pass FEL can be operated in the SR regime, where the radiation power is proportional to the square of the number of electrons^18^. Such SR pulses were examined in a single-pass FEL seeded by a Ti:sapphire laser by using a frequency-resolved optical gating technique^19^. In the experiment, nonlinear pulse shortening and formation of weak ringing were observed. The SPARC and FERMI@elettra groups have reported SR pulses generated from a high-gain harmonic-generation (HGHG) FEL^20^ and a cascaded HGHG FEL^21,22^.

The generation of SR pulses is also possible in FEL oscillators. In early experiments of FELs, the spiking behaviour was observed in low-gain highly-saturated FEL oscillators^23,24^. Moore and Piovella conducted a numerical study for such FEL oscillators and revealed that the FEL oscillator can be operated in the SR regime when it is driven by electron bunches having a shorter length than the slippage length, which is the product of the resonant radiation wavelength and the number of undulator periods, *N*_w_^25^. In such FEL oscillators, an optical pulse emitted in the slippage region interacts with new electron bunches entering the oscillator on every round trip and develops into a large peak intensity proportional to $N_{{\text{e}}}^{{2}}$ with efficiency scaling as $\sqrt{{N}_{\mathrm{e}}}$, where *N*_e_ is the number of electrons contributing to the lasing, the electrons in the bunch multiplied by the cavity Q value. Ultra-short pulse generation is another important feature of SR in FEL oscillators. Moore and Piovella’s research^25^ showed the generation of few-cycle pulses exhibiting ringing structures, similar to BC ringing of Dicke’s SR. Piovella et al. further conducted analytical studies and revealed that the FEL pulse duration is inversely proportional to the extraction efficiency^26,27^. These features distinguish the superradiant FEL oscillators from long-bunch FEL oscillators whose efficiency is limited by the number of undulator periods, η ~ 1/(2*N*_w_), and pulse duration is determined by the electron bunch length. The first demonstration of SR in an FEL oscillator was reported by Jaroszynski et al.^28^. They confirmed the *N*_e_^2^ dependence of the peak power in an FEL oscillator, FELIX. There have been several analytical and numerical studies on SR in FEL oscillators to discuss few-cycle pulse generation with BC ringing^27,29–31^. Measurements of SR pulses in FEL oscillators have also been conducted but are limited to interpretation of autocorrelation waveforms without phase recovery^28,32–34^ or to reconstruction of a pulse with coarse resolution^35^.

Here, we report the first full characterization of SR pulses generated from an FEL oscillator. The structure of the FEL pulses was precisely evaluated via a fringe-resolved autocorrelation measurement and a phase retrieval analysis. As a result, an SR pulse containing multiple subpulses with π-phase jumps, which is similar to an SR pulse from a two-level atomic system, was observed. The observed pulse structures, i.e., the intensity and the phase, were well reproduced by one-dimensional numerical simulations. The numerical results indicate that the sub-pulses with π-phase jumps originates from repeated formation and deformation of microbunches across successive bunch slices and superluminal evolution of the pulse in the FEL oscillator.

## Results

The experiments were carried out at KU-FEL, a mid-infrared FEL facility at Kyoto University^36^. KU-FEL has two operation modes of the electron gun: thermionic cathode operation and photocathode operation. For the lasing wavelength of approximately 11 μm, the efficiency of energy extraction from the kinetic energy of the electron beam to the FEL at the end of the macropulse was 5.5 and 9.4% for thermionic and photocathode operation, respectively^37,38^. Under these two operation modes, the temporal structure of FEL pulses was precisely retrieved from simultaneously obtained linear autocorrelation (LA) and nonlinear fringe-resolved autocorrelation (FRAC) traces. While FRAC is widely used for optical pulse measurements, it does not directly provide the optical phase information to reconstruct the pulse shape. For precise reconstruction of the FEL pulse structure, i.e., recovery of the intensity and phase, we adopted the “Evolutionary Phase Retrieval from Interferometric AutoCorrelation (EPRIAC)” algorithm^39^, in which the frequency spectrum of FEL pulses deduced by Fourier transformation of the LA trace is used along with the FRAC trace for the reconstruction.

### Pulse structure and phase of the FEL pulse under the thermionic mode

**Figure 1:**
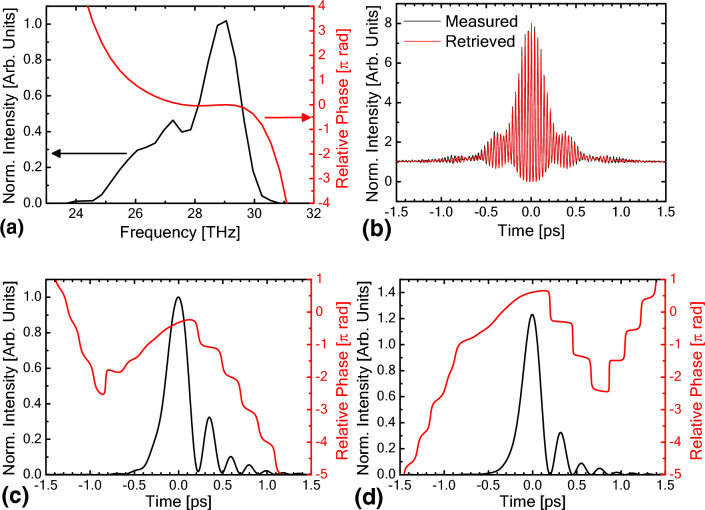
(**a**) FEL spectrum deduced by Fourier transformation of the LA trace and retrieved relative spectral phase. (**b**) Measured and retrieved FRAC traces. (**c**) Retrieved pulse structure and relative phase. (**d**) Retrieved pulse structure and relative phase after subtraction of the dispersion effect of the refractive optical components in the beam path.

Figure 1 shows the pulse measurement results for the average of a 270 ns time window around the peak of the FEL macropulse in the thermionic cathode mode with an extraction efficiency of 5%. In the measurement, an FEL pulse at the end of a macropulse was obtained by gating the signals. Figure 1a shows the frequency spectrum deduced from an LA trace and the spectral phase retrieved by EPRIAC. The measured and retrieved FRAC traces are shown in Fig. 1b. The measured FRAC traces can be well reproduced by the retrieved phase. The retrieved pulse shape and the relative temporal phase are shown in Fig. 1c. We removed the direction-of-time ambiguity in EPRIAC by observing the pulse elongation when a 10-mm-thick ZnSe window with a group velocity dispersion (GVD) of − 1365.9 fs^2^/mm at 10.3 μm was inserted into the beam path. The details of the pulse-direction determination are explained in Supplementary Material S2.

Into the FEL transport line from the out-coupling hole of the FEL optical cavity to the autocorrelation apparatus, two KRS-5 windows with a total thickness of 7 mm and one KBr window with a thickness of 3 mm were inserted. In addition, the autocorrelation apparatus had a ZnSe beam splitter with an effective thickness of 3.1 mm, and each beam of the autocorrelator passed through it once before hitting the second harmonic generation (SHG) crystal. In the few-cycle pulse measurements, the group delay dispersion (GDD) of these refractive optical components cannot be neglected. Since the relative phase distribution of the FEL pulse in the frequency domain was retrieved, the influence of these refractive optical components can be subtracted from the retrieved phase, and we can obtain the precise structure of the FEL pulse immediately after the out-coupling hole of the FEL optical cavity. The details of the subtraction of the influence of the refractive optical components are described in Supplementary Material S3. The reconstructed pulse shape and phase immediately after the out-coupling hole are shown in Fig. 1d. The pulse duration of the main pulse was approximately 230 fs in full width at half maximum (FWHM), which is equivalent to 6.7 optical cycles of 10.3-μm radiation. The relative phase has sudden π-phase jumps between pulses.

### Pulse structure and phase of the FEL pulse under the photocathode mode

**Figure 2:**
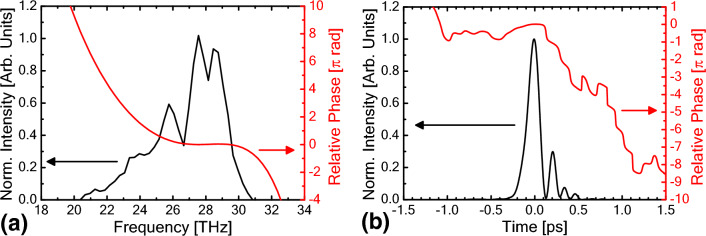
(**a**) Measured FEL spectrum and spectral phase. (**b**) Retrieved pulse shape and temporal phase of the FEL pulse generated at KU-FEL under the condition having an energy extraction efficiency of approximately 9%.

With the same procedure, the FEL pulse under photocathode operation with an energy extraction efficiency of approximately 9% was retrieved. The evaluated pulse shape and phase of the FEL pulse at the out-coupling hole are shown in Fig. 2b together with the measured frequency spectrum and the retrieved phase distribution at the out-coupling hole (Fig. 2a). The pulse duration of the main pulse was approximately 150 fs in FWHM and is equivalent to 4.2 optical cycles of 10.7-μm radiation (peak frequency of 28 THz). In this result, π-phase jumps were also observed. The FEL spectrum shown in Fig. 2a spans from 20 to 30 THz, which is much wider than that for the thermionic operation case (24.5–30 THz, shown in Fig. 1a).

### Comparison with numerical simulation

**Figure 3:**
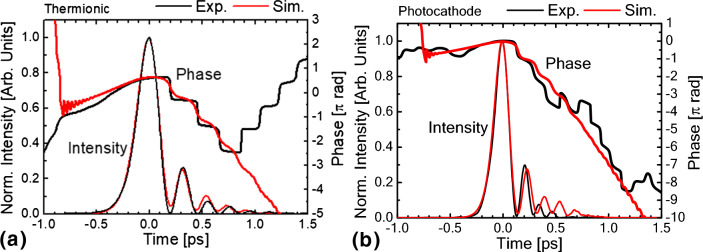
Comparison with 1D numerical simulation results. (**a**) Thermionic operation. (**b**) Photocathode operation.

In Fig. 3, the measured pulse structures and phase are plotted together with those obtained by numerical simulations. In both thermionic and photocathode operation, the intensity and phase of the main pulse obtained in the experiments are well reproduced by the numerical simulations. In the case of thermionic operation, the intensity and phase of the subpulses also show good agreement between the experimental and numerical results. In the case of photocathode operation, the numerical results have some discrepancies in the structure of the subpulses. The reasons for the discrepancies are discussed in the following section. Note that compensation of the influence of the refractive optical components in the beam path and the autocorrelation apparatus, which requires information on the spectral phase distribution, is indispensable for comparing the numerical results and experimental results.

## Discussion

**Figure 4:**
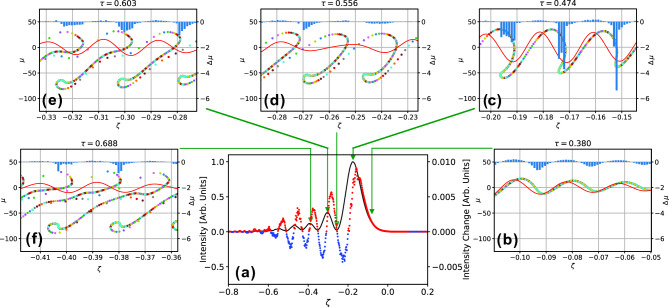
(**a**) Simulated FEL pulse at the end of the macropulse for the photocathode mode and local intensity changes in a single round trip, including the cavity loss of 3%, with red dots for amplification and blue dots for reduction. (**b**)–(**f**) snapshots representing the macroparticle distribution at different positions in the undulator for a specific bunch slice. The red lines in the snapshots indicate the electric field of the FEL pulse, and the blue bars are local changes in the macroparticle energy. The longitudinal variable, ζ = (*z − ct*)/*L*_s_, is defined in a coordinate system moving at the vacuum speed of light *c* and normalized by the slippage distance *L*_s_.

The formation of subpulses and π-phase jumps in the FEL pulses are similar to Burnham-Chiao ringing or SR ringing in two-level atomic systems^13^, which is coherent many-body Rabi oscillations originating from the light-matter resonant interaction. We discuss the mechanism of ringing formation in the FEL pulse with the help of numerical simulations. In Fig. 4, we plot the simulated FEL pulse at the end of the macropulse for the photocathode mode together with snapshots of the phase-space distributions. The FEL was in the saturation regime but not fully saturated at the end of the macropulse. In the plots, the longitudinal variable, ζ = (*z *− *ct*)/*L*_s_, is defined in a coordinate system moving at the vacuum speed of light *c* and normalized by the slippage distance *L*_s_, the time for traversing the undulator is normalized by the undulator length, *L*_u_, as τ = *ct*/*L*_u_, and the dimensionless electron energy is defined as μ = (Δ*E *− *E*_0_)/4π*N*_u_, where Δ*E* is the energy deviation from the resonance energy *E*_0_ and *N*_u_ is the number of undulator periods. Overlaid on the pulse shape, the local intensity changes in a single round trip, including the cavity loss of 3%, are indicated by red dots for amplification and blue dots for reduction. The snapshots represent the macroparticle distribution at different positions in the undulator for a specific bunch slice, whose initial position is ζ = 0.3 at τ = 0. The green arrows indicate the position of the slice in the FEL pulse when the snapshots were taken. In each snapshot, the electric field of the FEL pulse is plotted with a red line, and local changes in the macroparticle energy are plotted with blue bars. In the snapshots, the number of macroparticles was reduced to improve visibility.

As shown in Fig. 4, the radiation at the rising slope of the main pulse is amplified. In contrast, the radiation is reduced due to negative net gain at the falling slope of the first pulse. For the successive subpulses, amplification and reduction occur at the rising and falling slopes, respectively, in a similar manner.

To clarify the origin of the periodic gain modulation, we follow the motion of the macroparticles in the phase space. In the early part of the undulator (τ = 0.380, Fig. 4b), the bunch slices of interest are located at the rising slope of the first pulse, and the FEL electric field induces electron energy modulation. As the slices slip backwards in the FEL pulse, the energy modulation grows into a density modulation, forming microbunches with an interval equal to the FEL wavelength. At the peak of the main pulse (τ = 0.474, Fig. 4c), the electrons in the microbunch lose their energy as they emit strong radiation and thus slip backwards at a faster speed, stretching the phase distribution. At the dip between the main and second pulses (τ = 0.556, Fig. 4d), the density modulation almost disappears, and there is little energy exchange between the electrons and the radiation field. After passing the dip, the bunch slices again induce density modulation, emitting radiation for the second pulse (τ = 0.603, Fig. 4e). Note that the density modulation for the second pulse is established by the overlap of bunch slices over two radiation wavelengths. The third pulse is formed in a similar way by density modulation induced by slices over three radiation wavelengths (τ = 0.688, Fig. 4f). At the trailing edge of each pulse, the envelope of the radiation electric field goes through zero and becomes negative at the leading edge of the next subpulse. This reversal of the sign of the field envelope is the origin of the π-phase jumps. A movie file was prepared to visualize the macroparticle motion in the phase space. See Supplementary Video 1.

Through the above processes, the electrons form microbunches and transfer their energy to the radiation field with high conversion efficiency. Such efficient interaction is possible due to the FEL pulse with a well-designed temporal profile of the intensity and the phase, which is autonomously established after many round trips as an eigenmode of the SR FEL oscillator.

The ringing in the FEL pulse is analogous to the Burnham-Chiao ringing of two-level systems. The BC ringing is interpreted as an oscillation of the state vector on the Bloch sphere, in which phasing and dephasing of the macroscopic polarization of the atoms (or radiation and absorption of the light field by the atoms) occur alternately and the train of radiation pulses has π-phase jumps. The ringing in the FEL pulse originating from the repeated formation and deformation of microbunches is also accompanied by gain modulation and π-phase jumps.

The BC ringing with a π-phase jump in the light-matter resonant interaction is derived by solving the optical Bloch equations^13,40^. However, there have not been many experimental observations of BC ringing reported to date. Experiments of SR accompanied by BC ringing have been reported for HF gas^2^ and quantum dots in perovskite nanocrystals^41^, but π-phase jumps have not been examined there. π-phase jumps have been detected in the free-exciton transition in an optically thick semiconductor^42^ and the free induction decay in highly absorbing solutions^43^, both of which are a type of resonant light-matter interaction, although not SR. BC ringing with π-phase jumps was recently observed for the SR generated from lattice-confined atoms inside a hollow core fibre^44^.π-phase jumps are fundamental properties of SR pulses with BC ringing. Thus, the observation of π-phase jumps for the laser pulses generated from the FEL oscillator is strong evidence that the FEL oscillator operates in the SR regime. Our result is the first full characterization of an SR pulse with a subpicosecond duration.

As a result of the periodic gain modulation shown in Fig. 4, the FEL pulse is pushed forwards every round trip, and then, the group velocity of the pulse slightly exceeds the vacuum speed of light. This is known as superluminal propagation, which has been observed in various kinds of laser systems^45–48^. For FELs, there are theoretical studies on the superluminal propagation in oscillator FELs^31^ and seeded single-pass FELs^19,49^. In the present study, each sub-pulse is considered to have a superluminal property, and the complex pulse shape is preserved for many round trips due to the periodic modulation of the FEL gain.

The measured widths of the main pulses were 230 and 150 fs for thermionic operation with an extraction efficiency of 5% and photocathode operation with an extraction efficiency of 9%, respectively. The pulse width is almost inversely proportional to the extraction efficiency, as predicted by a theoretical study^27^. This is also evidence that the FEL operates in the SR regime.

There are two possibilities for further shortening of the FEL pulse duration. One is chirp compensation. As shown in Fig. 2, the FEL pulse is inherently down-chirped. By compensating for the linear chirp, the FEL pulse duration can be shortened down to 121 fs in FWHM in the case of photocathode operation, which corresponds to 3.4 optical cycles and is 16% longer than the transform-limited pulse (105 fs in FWHM and 2.9 optical cycles). See Supplementary Material S4 for more details. The other possibility is to increase the extraction efficiency by increasing the FEL gain or decreasing the loss of the optical cavity since the pulse duration of the FEL is almost inversely proportional to the efficiency. Experimental validation of these pulse shortening methods is planned at KU-FEL.

The numerical simulation results have discrepancies from the experimental results in the ringing structure. In the simulations, the gaps between the pulses are not zero, and therefore, the phase jumps are blunted. This can be attributed to the limitations of the simulation, such as the numerical diffusion in the finite-difference methods and one-dimensional approximation neglecting the transverse inhomogeneity of the electron and radiation beams. In the case of photocathode operation, the maximum energy decrease of electrons in the experiment was approximately 16%. This large energy change can induce transverse expansion of the electron beam in the latter part of the undulator and disturb the FEL interaction in the subpulses. Three-dimensional FEL simulations with unaveraged codes^50^ remain a future work.

## Methods

### Parameters and setup of KU-FEL

The experiments were carried out using the mid-infrared FEL oscillator at the KU-FEL facility^36^. The parameters under thermionic operation and photocathode operation are listed in Table 1. The electron bunch duration under the thermionic operation was characterized as 90–180 μm in FWHM at the electron beam energy of 27.5 MeV^51^. We expect that the bunch length at an electron beam energy of 28.5 MeV is the same as the reported value. The electron bunch duration under the photocathode operation has not been measured but expected to be shorter than the slippage length (556 μm) since high extraction efficiency (~ 9%) was obtained.

**Table 1:** Electron beam and undulator parameters.

Parameters	Thermionic	Photocathode
Injected electron beam energy	28.5 MeV	28.5 MeV
Electron bunch charge	55 pC maximum	~ 220 pC
Macropulse duration	6.8 μs	7 μs
Electron bunch repetition rate	2856 MHz	29.75 MHz
Undulator K-value	1.35	1.35
Undulator period length	33 mm	33 mm
Number of undulator periods	52	52
Energy extraction efficiency	~ 5%	~ 9%

The dynamic cavity desynchronization (DCD) method invented by the FELIX group^52^ has been implemented at KU-FEL to obtain high extraction efficiency with a short macropulse electron beam^37^. In each operation mode, DCD parameters, i.e., the timing and amount of frequency jump, were adjusted to obtain the highest output power under these operation modes. The energy extraction efficiencies for the two modes were evaluated from the differences in the electron energy with and without FEL oscillation^37^.

### Linear and nonlinear autocorrelation measurement

**Figure 5:**
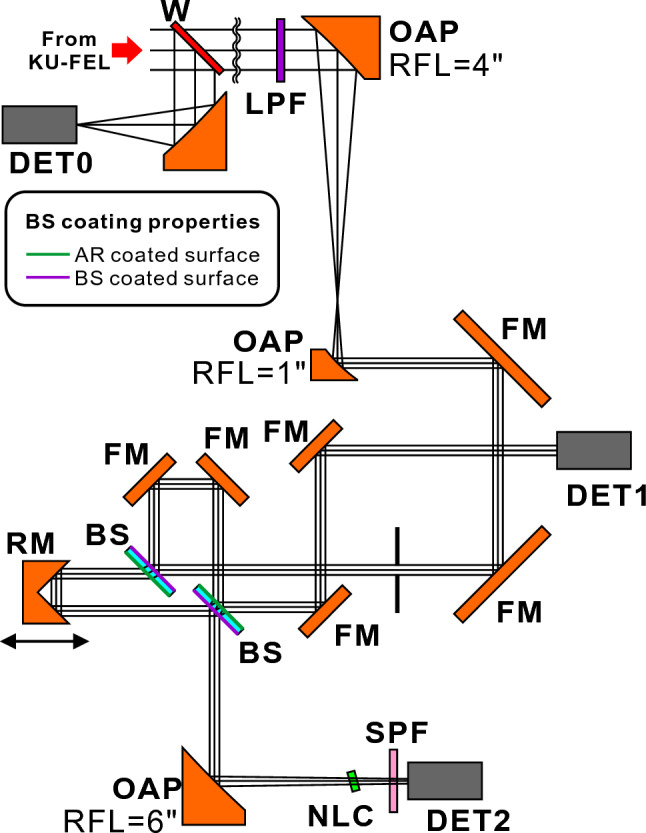
Schematic diagram of the autocorrelation setup for simultaneous acquisition of linear and nonlinear interferometric autocorrelation. LPF: longpass filter, SPF: shortpass filter, NLC: nonlinear crystal for SHG, BS: beam splitter, RM: roof mirror, OAP: off axis parabola, FM: flat mirror, W: KRS-5 window, DET0: detector for FEL power monitoring, DET1: detector for acquiring LA traces, and DET2: detector for acquiring nonlinear FRAC traces.

A schematic diagram of the autocorrelation setup for simultaneous acquisition of LA and FRAC traces is shown in Fig. 5. FEL oscillators inherently generate harmonics due to the higher-order frequency component of microbunched electron beams^53^. The harmonic components were removed by a longpass filter (LPF) with a cutoff wavelength of 7.3 μm (#68-656, Edmund Optics). A pair of off-axis parabolas (OAPs) with reflected focal lengths of 4 inches and 1 inch was used to reduce the FEL beam size by a factor of 4. Two beam splitters (BSs, #62-967, Edmund Optics) were used for splitting and combining the FEL beam. These two BSs, a roof mirror (RM) and two flat mirrors (FMs) were arranged to configure an interferometer. The RM was placed on a linear stage with a minimum step size of 0.5 μm (TSDM40-15X, Sigma-Koki) to change the temporal overlap condition of the split beams. These BSs have a beam-splitting coating and an antireflection coating. The front and rear surfaces of the BSs in the system were arranged as shown in Fig. 5. The split FEL beams injected into a nonlinear crystal (NLC) pass through the BS only once to reduce the influence of wavelength dispersion on the FRAC measurement. The split beams reaching detector 1 (DET1, Model 420, ELTEC Instruments, used for measuring the LA) pass through the BS twice, but the influence on the measured spectrum is negligible since the BS has flat spectral properties at the wavelength of interest. Before injection into the NLC, the combined beam was reflected by an OAP with a reflected focal length of 6 inches to make the beam size smaller to increase the SHG intensity. As the NLC, a AgGaSe_2_ crystal (8 × 8 × 0.5 mm, #APO-20-0410-01, 3photon) optimized for SHG at a wavelength of 10 μm was used. The thin crystal enables a wide phase-matching bandwidth. After the NLC, a shortpass filter (SPF) was arranged to block the fundamental component before injection into pyroelectric detector 2 (DET2, Model 420, ELTEC Instruments). DET1 and DET2 were connected to transimpedance amplifiers (TIA60, Thorlabs) to increase the signal intensity. The voltage signal outputs from the amplifiers were connected to an oscilloscope to record the signal intensity. Into the path of the FEL beam from the out-coupling hole of the optical resonator, an optical window made of KRS-5 with a thickness of 3 mm was inserted to reflect a part of the FEL light to monitor the intensity of the FEL by using a pyroelectric detector (DET0, Model 420, ELTEC Instruments) and a transimpedance amplifier (TIA60, Thorlabs). The voltage signal measured by DET1 was divided by the voltage signal measured by DET0. The voltage signal measured by DET2 was divided by the square of the voltage signal measured by DET0 since the SHG intensity depends on the square of the intensity of the injected FEL beam. These pyroelectric detectors combined with the transimpedance amplifiers have a frequency bandwidth of 30 MHz and can resolve the temporal evolution of the FEL macropulse (typically 2 μs and 4 μs in FWHM in the case of thermionic operation and photocathode operation, respectively). To measure the FEL micropulse structure at the end of the electron-beam macropulse, a time gate was arranged at the end of the electron-beam macropulse, and the average of the normalized signals was recorded. At each RM position, the signals of 4 macropulse shots were averaged to reduce the influence of shot-by-shot fluctuation.

### Retrieval of the spectral phase and time structure

From the measured linear and nonlinear autocorrelation traces, the spectral phase of the FEL pulse was retrieved based on the EPRIAC algorithm^39^. In this algorithm, the spectral phase is modelled with a Taylor expansion about the carrier frequency ω_0_.

$$\varphi \left( \omega \right) = \phi_{0} + \frac{{{\text{d}}\varphi \left( {\omega_{0} } \right)}}{{{\text{d}}\omega }}\left( {\omega - \omega_{0} } \right) + \frac{1}{2!}\frac{{{\text{d}}^{2} \varphi \left( {\omega_{0} } \right)}}{{{\text{d}}\omega^{2} }}\left( {\omega - \omega_{0} } \right)^{2} + \frac{1}{3!}\frac{{{\text{d}}^{3} \varphi \left( {\omega_{0} } \right)}}{{{\text{d}}\omega^{3} }}\left( {\omega - \omega_{0} } \right)^{3} + \cdots .$$

In this study, up to fifth-order terms were taken into account for modelling the FEL pulse. The first two terms represent the absolute phase and the group delay. Since they do not influence the pulse shape or its measurement, these two terms were neglected in the analysis. The second-order, third-order, fourth-order, and fifth-order coefficients were determined by an evolutionary algorithm. First, 200 genes with 4 elements corresponding to each coefficient were randomly generated. Then, the temporal profile of the complex electric field was calculated by Fourier transformation of the spectral amplitude calculated from the LA trace and spectral phase calculated from the coefficients. Then, an FRAC trace could be calculated from the temporal profile of the complex electric field. Here, 200 FRAC traces could be calculated from the 200 genes. The survival parameter of the *i-*th gene was calculated as

$${\text{S}}_{i} = \left[ {\mathop \sum \limits_{j}^{N} \left\{ {{\text{FRAC}}_{i} \left( j \right) - {\text{FRAC}}_{{{\text{meas}}}} \left( j \right)} \right\}^{2} } \right]^{ - 1} ,$$

where FRAC_*i*_(*j*) and FRAC_meas_(*j*) are the FRAC traces of the *i-*th gene and the measured result, respectively. A larger value of S_*i*_ implies a better fit between two FRAC traces. Using this survival parameter, all genes were ranked, and genes for the next generation were generated based on the evolutionary algorithm. In our analysis, 30 generations were examined for one set of initial conditions, and independent runs were repeated 200 times to avoid being trapped in a local maximum. Thus, 1.2 million genes were examined for one dataset. Finally, the genes that gave the highest survival parameter were selected from all runs and all generations. Then, the pulse structure and phase were calculated from the temporal profile of the complex electric field. Here, we should note that the frequency spectra calculated from the LA traces were filtered by ideal (rectangular) bandpass filters before the EPRIAC analysis and pulse structure retrieval since the LA traces contained widely distributed white noise over the entire frequency range. In the case of analysis of the thermionic operation results, the lower cutoff frequency and the higher cutoff frequency were selected as 23.7 THz and 30.6 THz, respectively, to reduce the influence of white noise. In the analysis of the photocathode operation results, the lower cutoff frequency and the higher cutoff frequency were selected as 20.4 THz and 30.6 THz, respectively.

The software packages for retrieving the spectral phase and calculating the pulse structure and phase were developed in the LabVIEW® environment. To test the software packages, a temporal profile of a complex electric field given by a 1-D numerical simulation of an FEL oscillator with 5% energy extraction efficiency was used. We confirmed that the developed software can retrieve the complex pulse shape and phase expected for a high-efficiency FEL oscillator (see Supplementary Material S1).

### FEL simulation

Simulations of the lasing behaviour of FEL oscillators are, in general, conducted by one- or three-dimensional codes based on macroparticle tracking^26,37^, in which the electron bunch is divided into many slices along the longitudinal direction and macroparticles are placed in each slice. The motion of the macroparticles is tracked based on the interaction with the optical and undulator fields, and the growth of the radiation field is obtained by averaging the phase-space parameters of the macroparticles over at least one radiation wavelength^54^. This type of simulation is called an averaged code. In the high-efficiency FEL oscillators studied in the present paper, the assumption that the macroparticles are fixed to a slice is not valid because the electron energy significantly changes through the undulator. Therefore, we adopted a one-dimensional unaveraged code, in which macroparticles are not confined to specific slices and may redistribute during propagation. The growth of the radiation field is obtained by superposing the electromagnetic fields created by individual macroparticles^55^.

In the simulations shown in Figs. 3 and 4, the slippage length, *L*_s_, was divided into 624 grid points. We assumed a parabolic electron bunch with a full width of 0.45*L*_s_ and an FEL gain parameter ρ = 0.0039 at the bunch centre for the thermionic cathode mode. For the photocathode mode, we assumed a Gaussian bunch with σ = 0.1*L*_s_ and ρ = 0.0061 at the bunch centre. The round-trip cavity loss was 3%, and the cavity length detuning was altered from *δL* =  − 0.0066*L*_s_ to *δL* = 0 at the 100-th round trip for the thermionic cathode mode and from *δL* =  − 0.015*L*_s_ to *δL* = 0 at the 30-th round trip for the photocathode mode. The number of macroparticles was 1440 for each bunch slice equal to the radiation wavelength.

## Supplementary Information

Supplementary Information 1.

Supplementary Information 2.

Supplementary Video 1.

## Data Availability

All the relevant data that support the findings of this study are available from the corresponding authors upon reasonable request.
