# Author Correction: Full characterization of superradiant pulses generated from a free-electron laser oscillator

**DOI:** 10.1038/s41598-023-35959-y

**Published:** 2023-06-01

**Authors:** Heishun Zen, Ryoichi Hajima, Hideaki Ohgaki

**Affiliations:** 1 Institute of Advanced Energy, Kyoto University, Gokasho Uji, Kyoto 611‑0011 Japan; 2 National Institutes for Quantum Science and Technology, Kizugawa, Kyoto 619‑0215 Japan

Correction to: *Scientific Reports* 10.1038/s41598-023-33550-z, published online 18 April 2023

The original version of this Article contained an error in Figure 3 where the line colour of the experimental results and the line colour of the simulation results were swapped in panel (a). The original Figure 3 and accompanying legend appear below.

**Figure 3:**
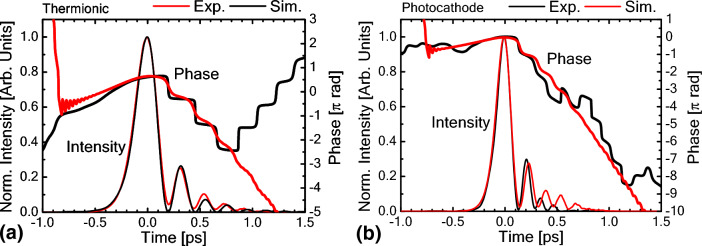
Comparison with 1D numerical simulation results. (a) Thermionic operation. (b) Photocathode operation.

The original Article has been corrected.

