# Supplementary material for: Full characterization of superradiant pulses generated from a free-electron laser oscillator
Source: Sci Rep. 2023 Apr 18;13:6350. doi: 10.1038/s41598-023-33550-z (PMC10113263; doi:10.1038/s41598-023-33550-z)
Supplement: Supplementary file 1 — Supplementary Information 1. [file 41598_2023_33550_MOESM1_ESM.docx]

Legend of Supplementary Video

Supplementary Video 1: A movie to represent the phase space motion of macroparticles in a simulation for the KU-FEL experiment with the photocathode mode. (Left panel): The dots are the macroparticles and the red line is the radiation field, which is defined so that the positive is the acceleration field. Local changes in the macroparticle energy are plotted as the blue bars with units at the right axes. (Right panel): The location of the macroparticles with respect to the FEL pulse is plotted by the red line.
