# Supplementary material for: Full characterization of superradiant pulses generated from a free-electron laser oscillator
Source: Sci Rep. 2023 Apr 18;13:6350. doi: 10.1038/s41598-023-33550-z (PMC10113263; doi:10.1038/s41598-023-33550-z)
Supplement: Supplementary file 2 — Supplementary Information 2. [file 41598_2023_33550_MOESM2_ESM.docx]

Supplementary Information for

**Full characterization of superradiant pulses generated from a free-electron laser oscillator**

Heishun Zen^1*^, Ryoichi Hajima^2^, Hideaki Ohgaki^1^

^1^Institute of Advanced Energy, Kyoto University, Gokasho Uji, 611-0011 Kyoto, Japan

^2^National Institutes for Quantum Science and Technology, Kizugawa, Kyoto 619-0215, Japan

*e-mail: zen@iae.kyoto-u.ac.jp

**S1. Examination of the developed software with a complex electric field obtained by a one-dimensional numerical simulation of an FEL oscillator with 5% energy extraction efficiency**

In this section, we describe the validation process for the software used for the free-electron laser (FEL) pulse measurements. The validation was completed by recovering a known pulse shape generated by a numerical simulation.

Figure S1(a) shows the complex electric field of an FEL pulse obtained by a one-dimensional numerical simulation of the FEL oscillator with 5% energy extraction efficiency and a carrier wavelength of 11.8 μm. The intensity and temporal phase of the FEL pulse are shown in Figure S1(b). This pulse contains multiple pulses and π-phase jumps, similar to the experimental results reported in the main text. From the complex electric field, the linear autocorrelation (LA) trace and the fringe-resolved autocorrelation (FRAC) trace of the FEL pulse were directly calculated and are shown in Figure S2. The obtained spectral distribution without phase information and the FRAC trace were used in the developed software to examine whether the software can accurately retrieve the pulse shape and phase. The original intensity and phase are compared with the retrieved intensity and phase in Figure S3. As can be seen in the figure, the complicated intensity and phase can be accurately retrieved by the developed software. In this examination, the pulse direction was used as the preknown parameter since the FRAC trace does not include information on the pulse front or tail. For actual measurement, a 10-mm-thick ZnSe plate was inserted into the beam path, and information on the pulse shape variation was used to determine the pulse direction, as described in the main text and Supplementary Information S2.

(a) (b)

Figure S1: (a) Complex electric field distribution obtained by a 1D numerical simulation. (b) Intensity and phase distribution calculated from the complex electric field distribution. The phase offset was adjusted to have the maximum phase be zero, and the large linear component of the phase evolution corresponding to the shift of the wavelength from the resonance wavelength used in the numerical simulation and calculation of the complex electric field distribution was also subtracted to display the π-phase jumps.

(a) (b)

Figure S2: (a) LA trace and (b) FRAC trace directly calculated from the complex electric field distribution obtained by a 1D FEL simulation.

Figure S3: Intensity and phase evolution of the original and retrieved pulses. The time offset and phase offset of the retrieved pulse were adjusted to obtain good matching with the original pulse.

**S2. Determination of the pulse direction by pulse shape measurement with insertion of a dispersive material**

Since the FRAC measurement result has a symmetric distribution over time, the pulse direction cannot be determined only from the FRAC measurement. In this study, we inserted a dispersive material (ZnSe, 10 mm thick, with an antireflection coating on both sides) into the FEL beam path, and the FEL pulse structures with and without the dispersive material were retrieved with the same method as the pulse shape measurement described in the main text. The measured FEL pulses with and without the ZnSe plate are shown in Figure S4. As can be seen in the figure, insertion of the ZnSe plate makes the FEL pulse duration longer. Since the group velocity dispersion of ZnSe at the FEL wavelength (10.3 μm in this experiment) is -1365.9 fs^2^/mm, the FEL pulse should have a frequency downchirp. The intensity and phase of the FEL pulse without the ZnSe plate are shown in Figure S5. In this case, the temporal phase of the main pulse has a negative second derivative coefficient. This implies that the main pulse has a downchirp. When the pulse direction is flipped, the sign of the temporal phase also flips. In the case of the flipped pulse direction, the measured result has a positive second derivative coefficient, i.e., a frequency upchirp. From the results measured with and without the ZnSe plate, the FEL pulse direction can be determined as the direction shown in Figure S5, i.e., smooth rising with a sech^2^-like temporal evolution and a successive ringing structure. The pulse direction and chirp direction are consistent with the 1D numerical simulation result (Figure S1(b)).

Figure S4: Measured FEL pulses without (black) and with (red) the ZnSe plate in the FEL beam path.

Figure S5: Intensity and phase of the FEL pulse without the ZnSe plate (same as Figure 1 in the main text).

**S3. Retrieval of the FEL pulse structure immediately after the coupling hole**

Since an ultrashort FEL pulse has a quite wide frequency spectrum, as shown in Figure 1(a) and 2(a), the pulse structure strongly depends on the dispersive materials in the FEL beam path. To obtain the original FEL pulse structure immediately after the out-coupling hole of the FEL cavity, the influences of the dispersive materials must be subtracted from the measured pulse structure. Since we can obtain the spectral phase distribution of the FEL pulse by Evolutionary Phase Retrieval from Interferometric AutoCorrelation (EPRIAC) [1] analysis, the FEL pulse structure immediately after the coupling hole can be retrieved by subtracting the phase variation due to the diffractive materials.

The refractive indices of the dispersive components in the beam path were calculated using the Sellmeier equations of the specific materials. In the experiment, there were KRS-5, ZnSe, and KBr windows in the beam path. The Sellmeier equations of these materials are given as [2]

(KRS-5) ${n_{KRS-5}(\lambda)}^{2}-1=\frac{1.8293958\lambda^{2}}{\lambda^{2}-0.0225}+\frac{1.6675593\lambda^{2}}{\lambda^{2}-0.0625}+\frac{1.1210424\lambda^{2}}{\lambda^{2}-0.1225}+\frac{0.04513366\lambda^{2}}{\lambda^{2}-0.2025}+\frac{12.380234\lambda^{2}}{\lambda^{2}-{27089.737}^{2}}$,

(ZnSe)　${n_{\mathrm{ZnSe}}(\lambda)}^{2}-1=\frac{4.45813734\lambda^{2}}{\lambda^{2}-{0.200859853}^{2}}+\frac{0.467216334\lambda^{2}}{\lambda^{2}-{0.391371166}^{2}}+\frac{2.89566290\lambda^{2}}{\lambda^{2}-{47.1362108}^{2}}$,

(KBr)　${n_{\mathrm{KBr}}(\lambda)}^{2}-1=0.39408+\frac{0.79221\lambda^{2}}{\lambda^{2}-{0.146}^{2}}+\frac{0.01981\lambda^{2}}{\lambda^{2}-{{0.173}^{3}}^{2}}+\frac{0.15587\lambda^{2}}{\lambda^{2}-{0.187}^{2}}+\frac{0.17673\lambda^{2}}{\lambda^{2}-{60.61}^{2}}+\frac{2.06217\lambda^{2}}{\lambda^{2}-{87.72}^{2}}$,

where *λ* is the wavelength in the unit of micrometres.

The relative difference in the optical phase shift Δ*φ*_A_ induced by dispersive material A with thickness *L* at the frequency of interest *f* from the reference frequency *f*_0_ can be calculated as

$$\Delta\phi_{A}\left( f-f_{0}, L \right)=-2\pi\frac{fn_{A}\left( \frac{c_{0}}{f}\times{10}^{6} \right)-f_{0}n_{A}\left( {\frac{c_{0}}{f}}_{0}\times{10}^{6} \right)}{c_{0}}L$$

because of the theoretical expression of a plane wave in a dispersive medium

$$E\left( f,z,n \right)=C\cos\left( 2\pi f\left( t-\frac{n}{c_{0}}z+\phi_{0} \right) \right),$$

where *z* and *φ*_0_ are the propagation distance in the dispersive medium and the initial phase before entering the dispersive medium, respectively. This phase shift was calculated for all frequencies and subtracted from the spectral phase distribution obtained by EPRIAC. Then, the pulse shape was obtained by performing complex inverse Fourier transformation using the obtained spectral phase distribution and original intensity distribution. Since the linear component of the spectral phase distribution in the frequency domain, i.e., the group delay, does not change the pulse shape, the linear component was omitted in the calculation results for simplicity.

The above treatment was validated by the experimental results obtained for pulse direction determination shown in Supplementary Information S2. The influence of the 10-mm-thick ZnSe plate was numerically subtracted from the experimental result obtained with the ZnSe plate. The result is shown in Figure S6. As shown in the figure, by subtracting the phase difference distribution due to the ZnSe plate, we can obtain the same pulse shape as that measured without the ZnSe plate.

To retrieve the FEL pulse structure at the out-coupling hole, the phase difference distributions for two KRS-5 plates with a total effective thickness of 7 mm, a ZnSe beam splitter with a total effective thickness of 3.1 mm, and a KBr plate with a thickness of 3 mm were subtracted from the experimental results.

Retrieval of the original pulse shape is important for discussing the detailed characteristics of the FEL pulse and for comparison with the numerical simulation results.

Figure S6: Measured pulse shape with (red) and without (grey) a 10-mm-thick ZnSe plate. The black line represents the pulse shape numerically retrieved by subtracting the influence of the 10-mm-thick ZnSe plate.

**S4. Expected FEL pulse duration with linear chirp compensation and perfect chirp compensation under photocathode operation**

As shown in Figure 3 in the main text, the superradiance FEL pulses inherently have a frequency downchirp. The FEL pulse duration can be further shortened by chirp compensation. Linear and perfect chirp compensation can be numerically performed since the spectral intensity and phase were obtained by the measurement. The linear and perfect chirp compensation results are shown in Figure S7. In the case of linear chirp compensation, the pulse duration of the main pulse can be shortened down to 121 fs in full width at half maximum (FWHM) (3.4 optical cycles at 28 THz). Even with linear chirp compensation, ringing remains after the main pulse. When the chirp is perfectly compensated, the ringing disappears, and the pulse duration is shortened down to 105 fs in FWHM (2.9 optical cycles at 28 THz).

Figure S7: Result of linear and perfect chirp compensation together with the original distribution. (a) Spectral intensity and phase distribution. (b) Pulse structure.

**S5. One-dimensional FEL simulation code**

In this section, we describe details of the simulation code used in the present study and calculation results that could not be included in the main text.

The generation of coherent radiation in an FEL can be calculated by numerically solving the Maxwell equation coupled with equations to derive the motion of electrons in the radiation and undulator magnetic fields. Since the number of electrons in a bunch, ~10^9^ for a 200 pC bunch, is too large, so-called macroparticles are introduced to conduct simulations with reasonable computer resources. In general FEL simulation codes, the electron bunch is divided into many slices along the longitudinal axis, and macroparticles are prepared in each slice. The motion of the macroparticles within a bunch slice is then tracked by considering the energy exchange between the electron and the radiation field. The evolution of the radiation electric field is calculated from the local driving source, i.e., the Fourier component of the beam current corresponding to the radiation wavelength. The Fourier component is obtained by averaging the contribution of macroparticles over at least one radiation wavelength. The simulation is, therefore, called the averaged code.

Averaged simulations, both one-dimensional and three-dimensional, have been widely used in the analysis of FELs. The averaged code is, however, not appropriate for the analysis of infrared FEL oscillators with large extraction efficiency because the assumption that the macroparticles are fixed to a slice is not valid for a high-efficiency FEL oscillator, in which some of the electrons significantly change energy and move across bunch slices through the undulator. Therefore, we adopted a one-dimensional unaveraged code, in which macroparticles are not confined to specific slices and may redistribute during propagation.

Following the paper that proposed the unaveraged FEL simulation [3], we modified our one-dimensional simulation code previously developed for analysis of FEL oscillators [4]. In the simulation code, the longitudinal variable is normalized as ζ=(*z*–*ct*)/*L*_s_, the time for traversing the undulator is normalized as τ= *ct*/*L*_u_, and the electron energy is normalized as μ=(Δ*E*–*E*_0_)/4π*N*_u_, where *N*_u_ is the number of undulator periods, *L*_s_=*N*_u_λ is the slippage distance, and Δ*E* is the electron energy deviation from the resonance energy *E*_0_. Macroparticles are prepared along the longitudinal coordinate ζ by reflecting the electron bunch profile, and the particles can move freely along ζ while passing through an undulator. The growth of the radiation field is updated every time step by superposing the electromagnetic fields created by individual macroparticles.

As discussed in paper [3], the effects of coherent spontaneous emission (CSE) appear explicitly in the unaveraged simulations. The CSE has another impact on the simulations of the high-efficiency FEL oscillator because the CSE increases the amplitude of the effective shot noise, which plays an essential role in both initiating the lasing and maintaining the lasing after saturation at the perfectly synchronized cavity length [5].

Figures S8 and S9 show the results of the unaveraged simulation with the same parameters as those used for the simulations of the photocathode mode operation presented in the main text. In these figures, the evolution of the FEL macropulse and pulse shapes for the 10^th^, 50^th^, 100^th^ and 250^th^ round trips are plotted. The normalized radiation intensity, |A|^2^, is defined such that ρ|A|^2^ gives the ratio between the energy density of the radiation and the resonant electron beam [6]. In Figure S9, continuous narrowing of the main pulse and the superluminal property can be identified. The distribution of the macroparticles in the longitudinal phase space at the end of the 210^th^ round trip is plotted in Figure S10, which depicts deformation of the bunch envelope due to the large drift of macroparticles in addition to the formation of microbunches whose interval is equal to the radiation wavelength.


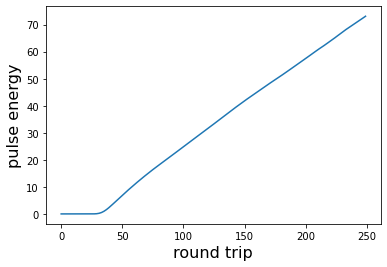

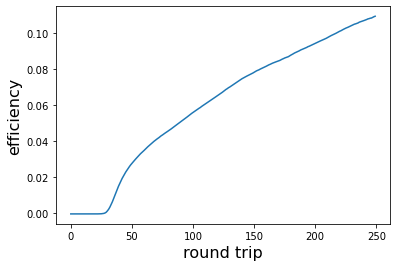


(A) (B)

Figure S8: Evolution of a macropulse calculated for photocathode mode operation. (A) FEL pulse energy and (B) extraction efficiency as a function of the round trip number.


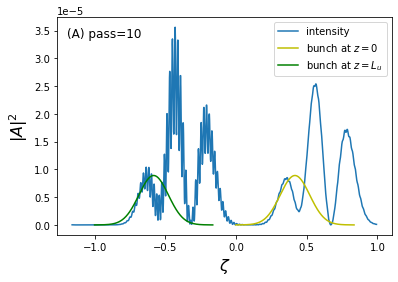

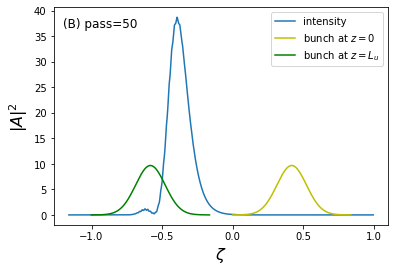


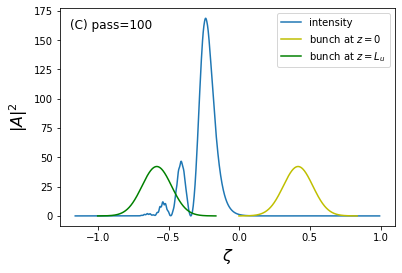

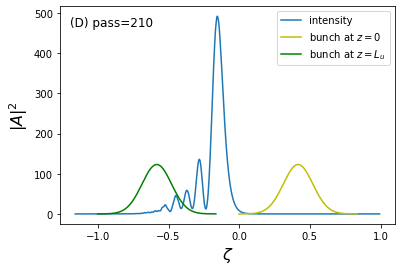


Figure S9: FEL pulse shapes for the (A) 10^th^, (B) 50^th^, (C) 100^th^ and (D) 250^th^ round trips calculated by the unaveraged code.


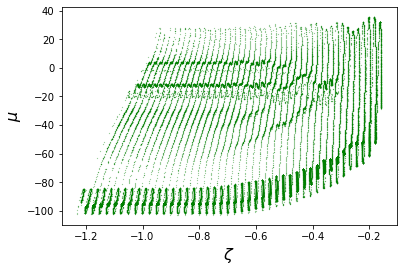


Figure S10: Distribution of the macroparticles at the end of the 210^th^ round trip.

Supplementary Video 1 (please see the “SupplementaryVideo1_1.mp4”): A movie to represent the phase space motion of macroparticles in a simulation for the KU-FEL experiment with the photocathode mode. (Left panel): The dots are the macroparticles and the red line is the radiation field, which is defined so that the positive is the acceleration field. Local changes in the macroparticle energy are plotted as the blue bars with units at the right axes. (Right panel): The location of the macroparticles with respect to the FEL pulse is plotted by the red line.

[1] K. H. Hong, Y. S. Lee, and C. H. Nam, Opt. Commun., 271, 169 (2007).

[2] <https://refractiveindex.info/>

[3] B.W.J. McNeil, G.R.N. Robb and D.A. Jaroszynski, Opt. Comm. 165, 65-70 (1999).

[4] R. Hajima and R. Nagai, Phys. Rev. Lett 91, 024801 (2003).

[5] R. Hajima, Atoms 9, 15 (2021).

[6] R. Bonifacio, F. Casagrande, G. Cerchioni, L. De Salvo Souza, P. Pierini and N. Piovella, Rivista Del Nuovo Cimento Vol.13, No.9 (1990).
